# Impact of periodontitis on type 2 diabetes: a bioinformatic analysis

**DOI:** 10.1186/s12903-024-04408-1

**Published:** 2024-05-29

**Authors:** Xindi Wei, Xiaomeng Zhang, Ruiying Chen, Yuan Li, Yijie Yang, Ke Deng, Zhengzhen Cai, Hongchang Lai, Junyu Shi

**Affiliations:** 1 Department of Oral and Maxillofacial Implantology, Shanghai PerioImplant Innovation Center, Shanghai Ninth People’s Hospital, Shanghai Jiao Tong University School of Medicine; College of Stomatology, Shanghai Jiao Tong University; National Center for Stomatology; National Clinical Research Center for Oral Diseases, Shanghai Key Laboratory of Stomatology; Shanghai Research Institute of Stomatology, 639 Zhizaoju Road, Shanghai, 200011 China; 2 Division of Periodontology and Implant Dentistry, The Faulty of Dentistry, The University of Hong Kong, Hong Kong, 999077 China

**Keywords:** Periodontitis, Type 2 diabetes, Mendelian randomization, Genome-wide association study, Single cell analysis

## Abstract

**Background:**

Periodontitis is strongly associated with type 2 diabetes (T2D) that results in serious complications and mortality. However, the pathogenic role of periodontitis in the development of T2D and the underlain mechanism have not been fully elucidated.

**Methods:**

A Mendelian randomization (MR) was performed to estimate the causality between two diseases. Bioinformatics tools, including gene ontology and pathway enrichment analyses, were employed to analyze the common differentially expressed genes (DEGs) in periodontitis and T2D. MR and colocalization analyses were then utilized to investigate the causal associations between potential pathogenic gene expression and the risk of T2D. Single cell-type expression analysis was further performed to detect the cellular localization of these genes.

**Results:**

Genetically predicted periodontitis was associated with a higher risk of T2D (OR, 1.469; 95% CI, 1.117–1.930; *P* = 0.006) and insulin resistance (OR 1.034; 95%CI 1.001–1.068; *P* = 0.041). 79 common DEGs associated with periodontitis and T2D were then identified and demonstrated enrichment mainly in CXC receptor chemokine receptor binding and interleutin-17 signaling pathway. The integration of GWAS with the expression quantitative trait locis of these genes from the peripheral blood genetically prioritized 6 candidate genes, including 2 risk genes (*RAP2A*, *MCUR1*) and 4 protective genes (*WNK1, NFIX, FOS, PANX1*) in periodontitis-related T2D. Enriched in natural killer cells, *RAP2A* (OR 4.909; 95% CI 1.849–13.039; *P* = 0.001) demonstrated high risk influence on T2D, and exhibited strong genetic evidence of colocalization (coloc.abf-PPH4 = 0.632).

**Conclusions:**

This study used a multi-omics integration method to explore causality between periodontitis and T2D, and revealed molecular mechanisms using bioinformatics tools. Periodontitis was associated with a higher risk of T2D. *MCUR1*, *RAP2A*, *FOS*, *PANX1*, *NFIX* and *WNK1* may play important roles in the pathogenesis of periodontitis-related T2D, shedding light on the development of potential drug targets.

**Supplementary Information:**

The online version contains supplementary material available at 10.1186/s12903-024-04408-1.

## Background

Periodontitis, a significant contributor to the overall global disease burden, represents a public health problem [1]. Increased by 8.44% (6.62-10.59%) in just 20 years, the age-standardized prevalence rate of periodontitis reached 13,109 (9,993-16,385) per 100,000 people in 2019 [2]. Epidemiologic studies indicating a correlation between periodontitis and at least 43 systemic diseases, including type 2 diabetes (T2D) [3–7]. T2D is associated with serious complications and mortality, resulting in low quality of life and substantial socioeconomic burden [8]. Periodontitis patients have been reported to have a higher risk of T2D, with severe periodontitis increasing the risk of T2D by 53% [9–11]. Another study indicates that moderate or severe periodontitis is related to a higher risk of all-cause mortality in diabetes patients [12].

Previous studies indicated that periodontitis may contribute to T2D development due to systemic microinflammation [11, 13]. The microbiome associated with periodontitis may activate the host immune response, leading to the secretion and cascade amplification of numerous pro-inflammatory cytokines, thereby promoting the progression of T2D [14]. Moreover, immune cells exhibited similar functional alterations in T2D and periodontitis [15]. A notable pathway RESISTIN, which could increase insulin resistance and susceptibility to diabetes, was found to be activated under periodontitis conditions, potentially linking periodontitis to T2D [16]. Despite the findings of these studies, there remains limited research confirming causally that periodontitis can precipitate the onset of diabetes. Moreover, the specific molecular mechanisms through which the key pathogenic genes mediate periodontitis-related T2D remain largely ambiguous.

Mendelian randomization (MR) emerges as an alternative method to randomized clinical trial, allowing for the exploration of potential causality between an exposure and the outcome by employing use of genetic variants as instrumental variables (IVs) [17]. Similar to randomization in randomized clinical trial, genetic variants are randomly assigned during gamete formation and conception, thus minimizing the impact of confounders and reverse causation. The expression quantitative trait loci (eQTL) can reveal the associations of single nucleotide polymorphisms (SNPs) with level of gene expression. The increasing availability of eQTL data enables the exploration of causal association between gene expression and traits via MR [18], allowing the identification of key pathogenic genes in diseases.

In this study, a two-sample MR analysis is conducted to illustrate the relationship between periodontitis and T2D. Multiple bioinformatics tools are utilized to identify the differentially expressed genes (DEGs) and elucidate the potential mechanism underlying periodontitis-related T2D. Subsequently, MR is employed to explore the associations between pathogenic gene expression and the risk of T2D. Colocalization analysis is employed to provide additional evidence supporting causality. Lastly, single-cell expression analysis is conducted to investigate the cellular localization of these genes. Through these methodologies, this study aims to deepen the understanding of the role of periodontitis in the development of T2D, offering new perspectives for T2D management.

## Methods

### Overall study design

**Fig. 1:**
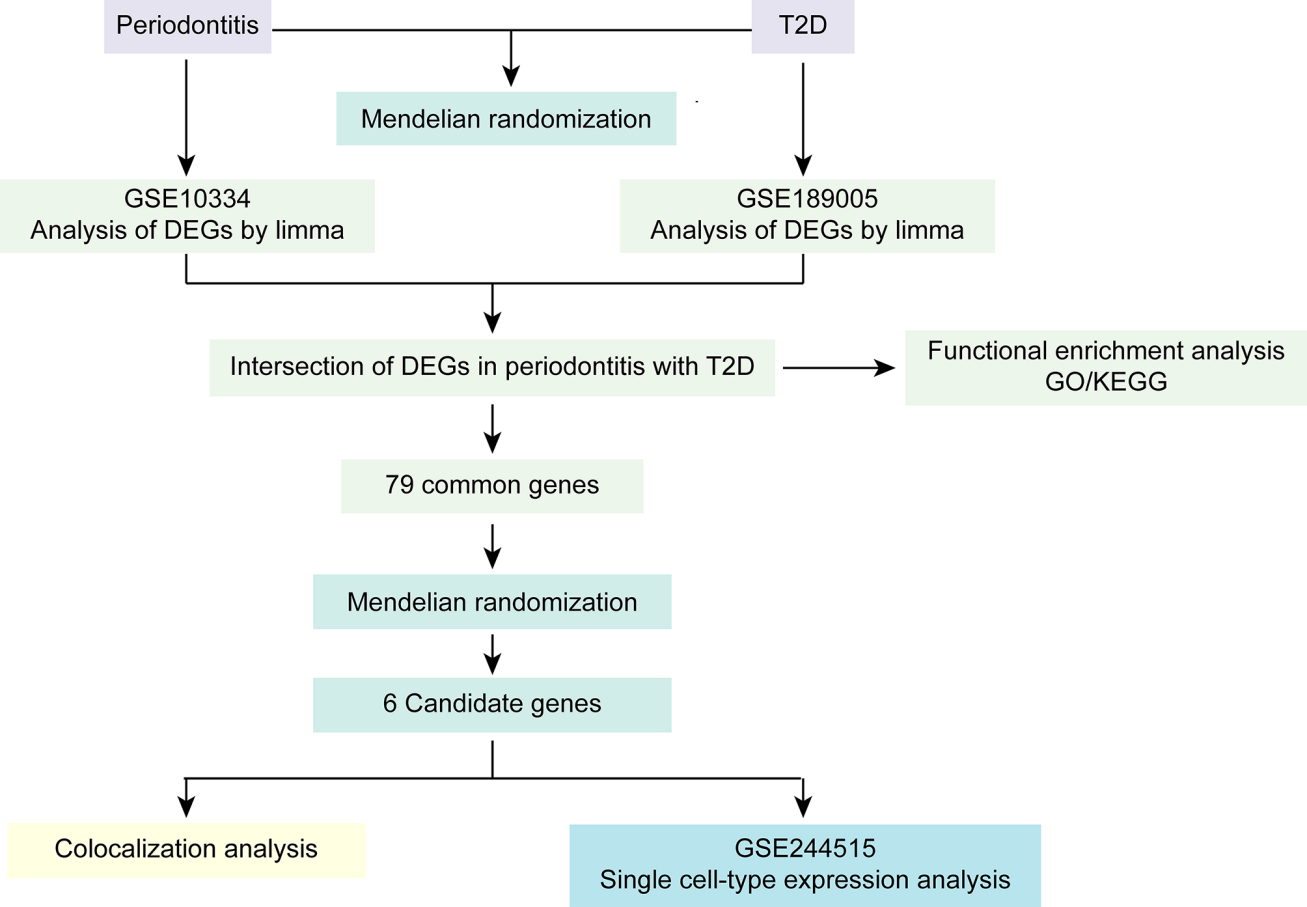
The framework of the study. DEGs indicates differentially expressed genes; T2D indicates type 2 diabetes

The framework of the study is depicted in Fig. 1. Briefly, two-sample MR was employed to explore the causal association between periodontitis and T2D. To discover the mechanisms of periodontitis-related T2D, common DEGs related to periodontitis and T2D were identified and analyzed using Gene Ontology (GO) and Kyoto Encyclopedia of Genes and Genomes (KEGG) functional enrichment analysis. Then, eQTL data were employed to examined the associations between DEGs expression levels and T2D using a MR framework. Colocalization analysis was leveraged to further verify the causal associations between the candidate genes and T2D. Last, single cell-type expression analysis was conducted to investigate the cellular localization of candidate genes in peripheral blood mononuclear cells (PBMCs) of patients with T2D and periodontitis.

### GWAS data sources of periodontitis, T2D and eQTL

For the analysis of periodontitis, the genome-wide association study (GWAS) summary statistics of periodontitis in European ancestry that includes 4,434 periodontitis cases and 259,234 controls from FinnGen were selected (Supplementary Table 1). Details of this project could be found in the FinnGen research project (https://www.finngen.fi/fi). The International Classification of Diseases, Tenth Revision (ICD-10) was used to define the periodontitis cases. The severity of periodontitis was not reported in the database. Regarding T2D, the GWAS summary statistics of Europeans were obtained from the DIAGRAM Consortium (http://www.diagram-consortium.org/) and the IEU OpenGWAS project (https://gwas.mrcieu.ac.uk/). European ancestry specific dataset from the DIAGRAM Consortium (180,834 T2D cases and 1,159,055 controls) was included in the discovery MR analysis [19], and T2D dataset from IEU OpenGWAS (4,040 T2D cases and 116,246 controls) was further analyzed in the validation MR. The GWAS statistics of the level of fasting insulin (FI) were obtained from the Meta-Analyses of Glucose and Insulin-related traits Consortium (MAGIC) (http://magicinvestigators.org/), which included 281,416 individuals without diabetes. All cited data sources obtained participant informed consent and relevant ethical approval. The cis-eQTL data from whole blood and PBMC tissues were extracted from eQTLGen Consortium (https://www.eqtlgen.org/) which included 14,263 individuals [20].

### Selection of genetic variants as IVs

The SNPs associated with periodontitis were selected at the genome-wide significance of *P* < 5 × 10^− 6^ [21]. The independence of the IV was ensured via the linkage disequilibrium (LD) clumping algorithm with the stringent cut-off r^2^ = 0.001. Proxy-SNPs were not used as IVs when there were no matching SNPs in the outcome data. To ascertain the independence assumption, SNPs were checked in PhenoScanner V2 (http://www.phenoscanner.medschl.cam.ac.uk/) to test if these SNPs were correlated with the potential cofounding factors [22]. Any SNPs found to be associated with any of the confounders were subsequently excluded from this study. F statistic was used to determine the power of the remaining SNPs, and the SNP with an F statistic < 10 was removed. The F statistic was associated with the proportion of variance in the phenotype explained by the genetic variants (R^2^), sample size (n) and number of instruments (k) and calculated by the formula (1) and (2) [23].

1 $$F=\frac{{\left(n-k-1\right)*R}^{2}}{{k(1-R}^{2})}$$

2 $${R}^{2}=2*EAF*\left(1-EAF\right)*{\beta }^{2}$$

### Mendelian randomization

The TwoSampleMR and MendelianRandomization packages were mainly used to perform MR analyses [24, 25]. The inverse-variance-weight (IVW) method was the primary method employed for analysis. Heterogeneity in IVW analysis was estimated by using the Cochran Q statistic. Specifically, a random-effects IVW MR analysis should be used if Cochran Q statistic indicates heterogeneity [26]. The presence of pleiotropy was estimated using MR-Egger regression based on its intercept term [27, 28]. The slope coefficient from MR-Egger regression was utilized for evaluating causal effect when pleiotropy was detected. The pleiotropy was also assessed through MR pleiotropy residual sum and outlier (MR-PRESSO) that is designed to identify horizontal pleiotropic outliers [29]. Weighted median, Simple mode and Weight mode analyses are performed as sensitivity analyses to bolster the robustness of the findings of IVW method [30, 31]. The sensitivity of SNPs was tested through leave-one-out analysis that performing multiple analyses after sequentially removing 1 SNP from the IVs set. Moreover, the impact of each SNP on the outcome was visualized in forest plots. The funnel plots were also used to assess potential biases in the results. The MR study adheres to the recommendations by the Strengthening the Reporting of Observational Studies in Epidemiology Using Mendelian Randomization (STROBE-MR) reporting guideline [32].

### Collection of periodontitis and T2D related genes

The microarray dataset consisted of 9 T2D whole-blood samples and 9 healthy whole-blood samples (GSE189005) and dataset consisted of 183 periodontitis site samples and 64 healthy periodontal site samples (GSE10334) were downloaded from the Gene Expression Omnibus (GEO) database (https://www.ncbi.nlm.nih.gov/geo/) [33]. The limma package was used to screen the DEGs in datasets [34]. DEGs were identified upon the thresholds of adjusted *P* value < 0.05 and |log2(fold change)|>0.5. Subsequently, the expression patterns of DEGs were visualized in the form of volcano plot and heatmap with the ggplot2 and pheatmap packages. The common DEGs of periodontitis and T2D were then overlapped for further analyses.

### Functional enrichment analysis

To understand the functional characteristic of the common DEGs in periodontitis and T2D, GO and KEGG pathway enrichment analysis were then conducted by ClusterProfiler package [35]. A threshold of *P* < 0.05 was defined as significant enrichment. The results of functional enrichment analysis were displayed via bar plot.

### Colocalization analysis

To assess whether causal gene and T2D risk were consistent with a shared causal variant, the colocalization analyses were conducted based on coloc and locuscomparer R packages [36, 37]. For each gene, SNPs within ± 1000,000 kb of the eQTL were included. When a gene had more than one eQTL, colocalization analysis was performed based on the eQTL with the most significant *P* value. The eQTL with the strongest evidence for colocalization was shown in the regional association plots. PPH4 > 0.6 was defined as the threshold for the shared genetic effects between the two traits [38].

### Single cell-type expression analysis

The cell type-specific expression of causal genes was further evaluated by employing single-cell data of PBMCs from patients with periodontitis and T2D (GSE244515) from GEO database [33]. Data preprocessing was carried out by Seurat and Harmony packages [39, 40]. The SingleR package was used to annotate the cell types [41], and the clustering results were visualized using uniform manifold approximation and projection (UMAP).

## Results

### Identification of causal association between periodontitis and T2D

**Fig. 2:**
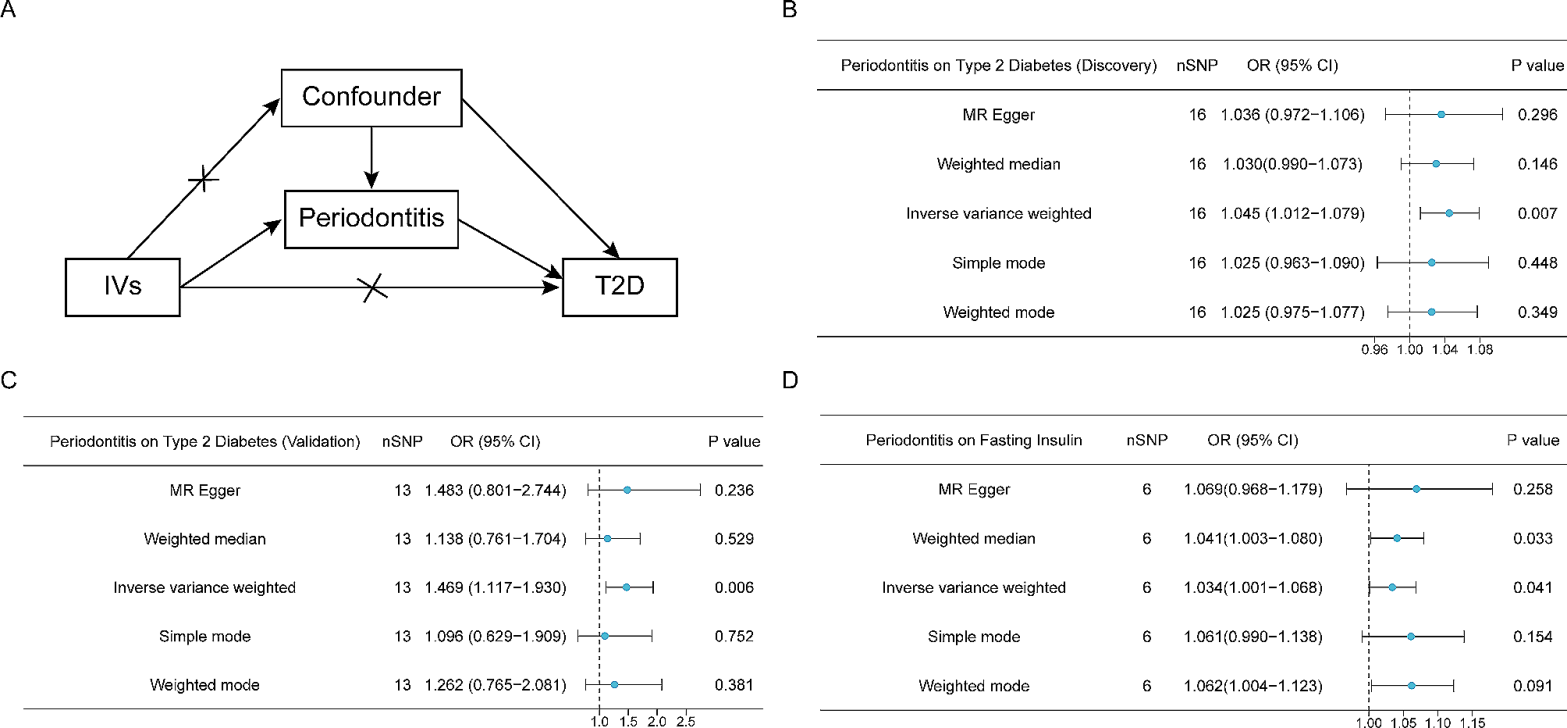
MR results indicate the relationship between periodontitis and T2D. (**A**) The basic principles of the MR study. (**B**) MR estimates for the impact of periodontitis on T2D (discovery phase). (**C**) MR estimates for the influence of periodontitis on T2D (validation phase). (**D**) MR estimates for the impact of periodontitis on FI. IV indicates instrumental variables; OR indicates odds ratio; CI indicates confidence interval. The error bars represent 95%CIs. All statistical tests were two-sided. *P* < 0.05 was considered significant

To explore the causality between periodontitis and T2D, MR analysis was performed (Fig. 2A). As presented in Supplementary Table 2, 17 independent significant SNPs nominally related to periodontitis were selected. IVW method was conducted as the primary analysis, demonstrating that T2D was causally associated with an increased risk of periodontitis (OR, 1.045; 95% CI, 1.012–1.079; *P* = 0.007). However, the MR-Egger, Weighted median, Simple mode and Weight mode analyses did not present significant associations between periodontitis and T2D (Fig. 2B). Sensitivity analyses revealed no evidence of heterogeneity (Q statistic = 18.89; *P* = 0.219) or pleiotropy (MR-Egger intercept *P* = 0.772). The MR-PRESSO global outlier test did not identify a significant impact of outliers (*P* = 0.265). Another statistics was analyzed to test the robustness of the conclusion. For this dataset, the IVW leave-one-out analysis showed that rs141098993 was an outliner. After excluding rs141098993, genetically predicted periodontitis was causally associated with a higher risk of T2D (OR, 1.469; 95% CI, 1.117–1.930; *P* = 0.006) (Fig. 2C), further substantiating the causal relationship. Since insulin resistance was an initial abnormality in the development of T2D, relationship between periodontitis and FI, the biochemical parameters that reflected the β-cell insulin production and insulin resistance, was further analyzed to confirm the conclusion [42, 43]. The IVW analysis results indicated that periodontitis was associated with increased level of FI (OR 1.034; 95%CI 1.001–1.068; *P* = 0.041) (Fig. 2D). The results were corroborated by the weight median method (OR 1.041; 95%CI 1.003–1.080; *P* = 0.033), indicating reliability of the conclusion. The IVW leave-one-out analysis, scatter plot, forest plot, and funnel plot did not show any leverage points with high influence (Supplementary Figs. 1–3).

### Functional enrichment of DEGs involved in periodontitis-related T2D

**Fig. 3:**
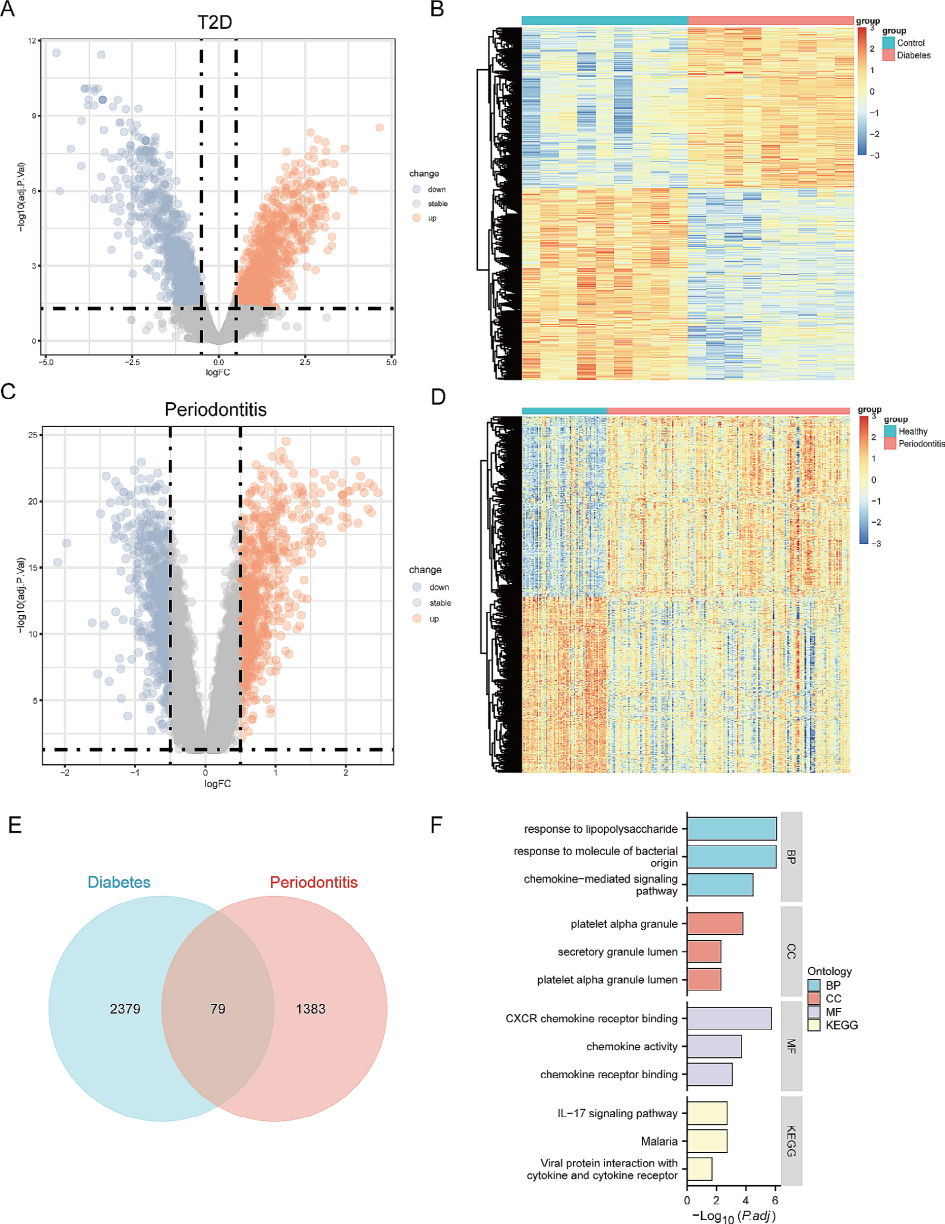
Identification of DEGs in periodontitis-related T2D, and followed by enrichment analyses. (**A**) The volcano plot revealing DEGs in the T2D dataset. (**B**) The heatmap representing the DEGs in the T2D dataset. (**C**) The volcano plot revealing DEGs in the periodontitis dataset. (**D**) The heatmap representing the DEGs in the periodontitis dataset. (**E**) The intersection of T2D DEGs with periodontitis DEGs via the Venn diagram. (**F**) The GO/KEGG enrichment analyses of pathogenic genes in periodontitis-related T2D. T2D indicates type 2 diabetes; PBMC indicates peripheral blood mononuclear cells; DEG indicates differentially expressed genes

To explore the genes involved in the pathogenesis of periodontitis-related T2D, expression profiles of periodontitis and T2D from the GEO database were analyzed. In the periodontitis dataset, 1,462 DEGs were collected with |Log2(fold change)|>0.5 and |adj.p.Val.|<0.05, and 2,458 DEGs were identified in the T2D dataset (Fig. 3A-D). Venn diagram revealed that 79 DEGs were involved in both periodontitis and T2D (Fig. 3E). The GO and KEGG enrichment analyses were performed to better understand the function and mechanism of the pathogenic genes. Biological process (BP) of GO term analysis demonstrated that the pathogenic genes in periodontitis-related T2D were mostly enriched in response to lipopolysaccharide and molecule of bacterial origin. In terms of cellular component (CC) of GO term analysis, the pathogenic genes were mainly located in platelet alpha granule and secretory granule lumen. For molecular function (MF) analysis, the results suggested that CXC receptor chemokine receptor binding and chemokine activity were the most relevant items of the pathogenic genes. KEGG pathway analysis indicated that the pathogenic genes in periodontitis-related T2D were associated with interleutin-17 (IL-17) signaling pathway and malaria (Fig. 3E).

### Identification of T2D causal genes

**Fig. 4:**
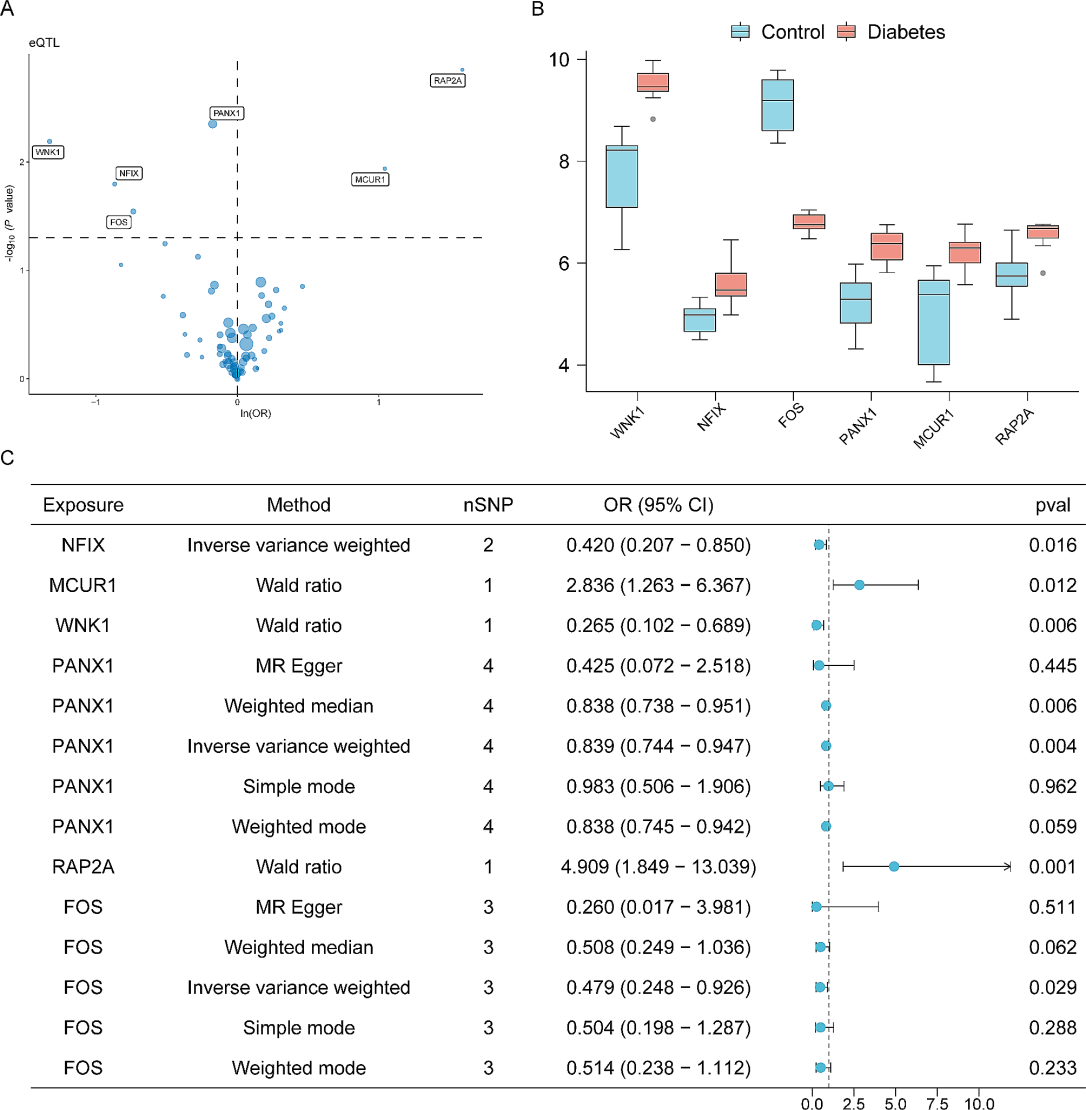
MR analyses results of T2D causal genes. (**A**) The volcano plot revealing 6 potential causal genes for T2D. (**B**) The box plot showing the expression levels of causal genes for T2D in T2D-PBMCs. (**C**) The forest plot for the MR results between blood eQTL and T2D. The error bars represent 95%CIs. All statistical tests were two-sided. *P* < 0.05 was considered significant

To further investigate the molecular mechanisms, eQTLs of the pathogenic genes from the blood were obtained to perform MR from eQTLGen Consortium. As a result, 6 potential candidate genes were identified to have causal associations with T2D (Fig. 4A). Mitochondrial calcium uniporter regulator 1 (*MCUR1*) (OR 2.836; 95% CI 1.263–6.367; *P* = 0.016) and RAS-related protein Rap-2a (*RAP2A*) (OR 4.909; 95% CI 1.849–13.039; *P* = 0.001) were up-regulated in T2D-PBMCs and demonstrated risk influence on T2D. FBJ murine osteosarcoma viral oncogene homolog (*FOS*) (OR 0.479; 95% CI 0.248–0.926; *P* = 0.029) was down-regulated in T2D-PBMCs, demonstrating protective influence on T2D. However, nuclear factor IX (*NFIX*) (OR 0.420; 95% CI 0.207–0.850; *P* = 0.016), pannexin 1 (*PANX1*) (OR 0.839; 95% CI 0.744–0.947; *P* = 0.004) and with no lysine-1 (*WNK1*) (OR 0.265; 95% CI 0.102–0.689; *P* = 0.006) were up-regulated in T2D-PBMCs and showed protective influence on T2D (Fig. 4B-C).

### Colocalization analyses of candidate genes

**Fig. 5:**
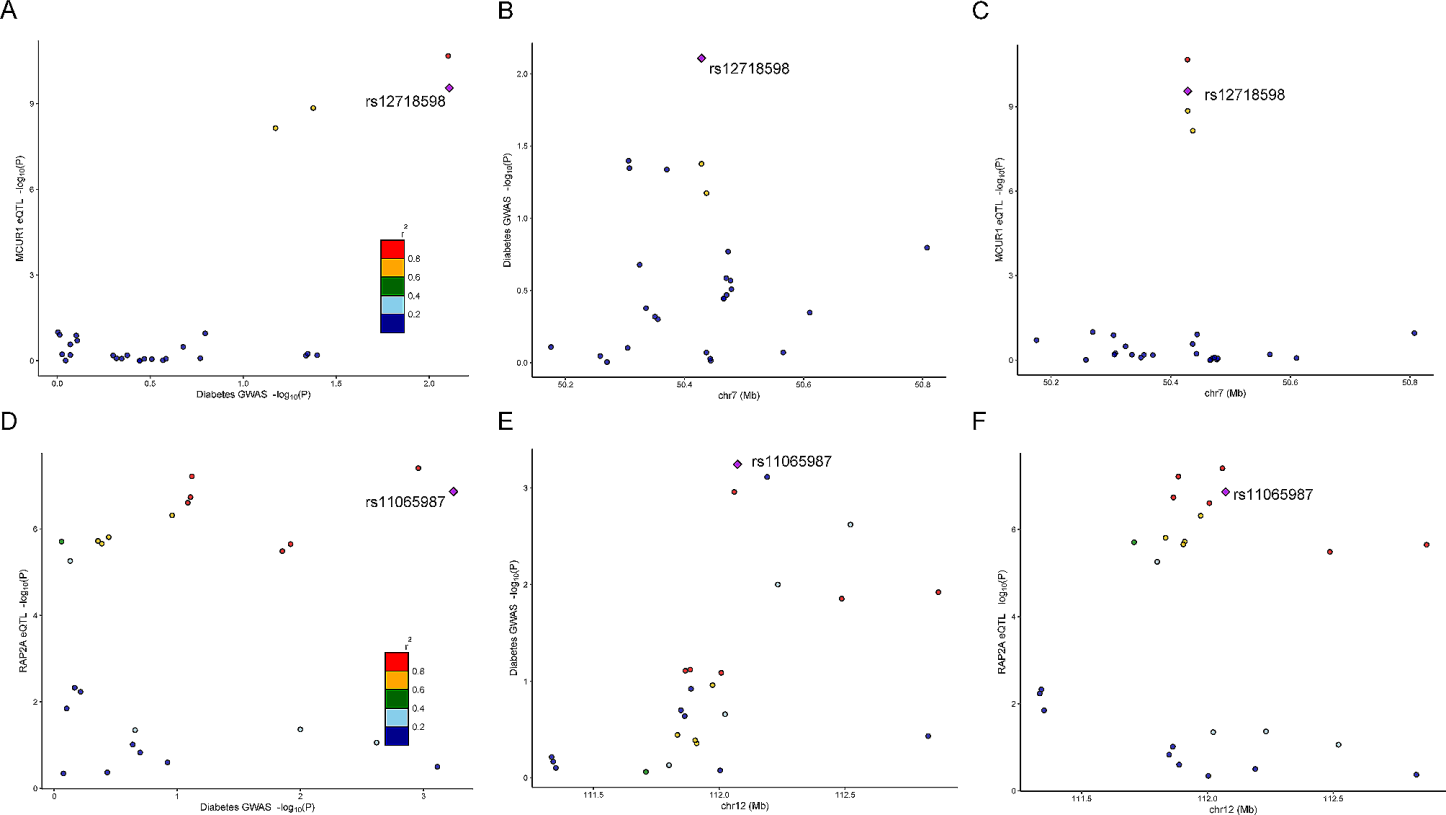
Colocalization analyses of *MCUR1* and *RAP2A*. (**A-C**) The regional association plot for colocalization analysis of *MCUR1*. (**D-F**) The regional association plot for colocalization analysis of *RAP2A*

Subsequently, GWAS-eQTL colocalization were analyzed (Supplementary Table 3). For the candidate genes, *RAP2A* (coloc.abf-PPH4 = 0.632) (Fig. 5A-C) supported strong evidence of genetic colocalization, while *MCUR1* (coloc.abf-PPH4 = 0.268) (Fig. 5D-F), *WNK1* (coloc.abf-PPH4 = 0.302), *NFIX* (coloc.abf-PPH4 = 0.259) and *PANX1* (coloc.abf-PPH4 = 0.372) demonstrated relatively weak colocalization evidence. *FOS* (coloc.abf-PPH4 = 0.159) presented almost no colocalization evidence (Supplementary Fig. 4).

### Cell-type specificity expression of causal genes in PBMCs

**Fig. 6:**
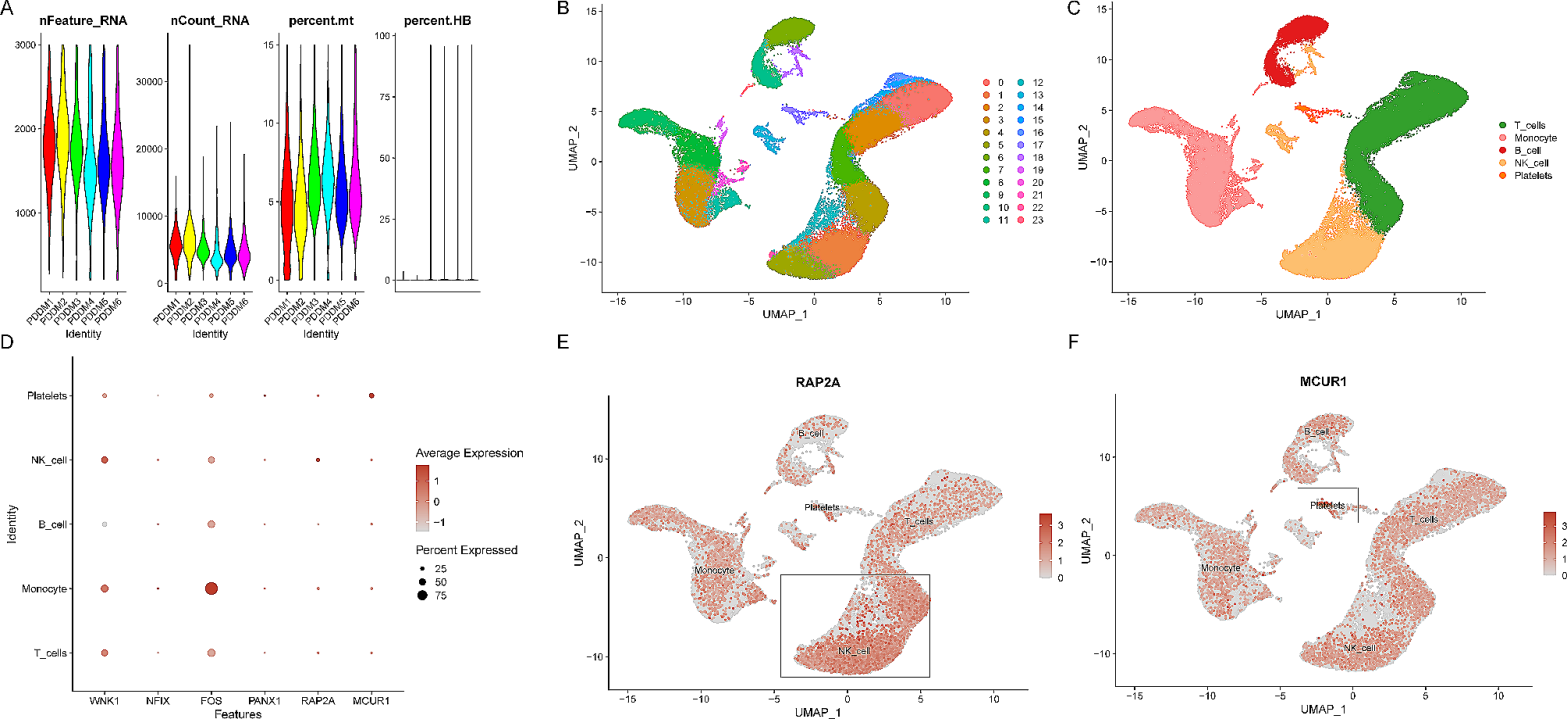
Single cell-type expression of *RAP2A* and *MCUR1* in PBMCs. (**A**) The violin plots showing the number of features, RNA counts, percent mitochondrial transcripts and percent hemoglobin found in PBMCs prior to quality control. (**B**) UMAP representation of 24 cell clusters identified by scRNA-seq. (**C**) UMAP representation of 5 cell types. (**D**) Dot plot depicting expression of candidate genes in each cell type. (**E**) The expression of *RAP2A* in each cell type. (**F**) The expression of *MCUR1* in each cell type

To explore whether candidate genes had cell type-specific enrichment in PBMCs of patients with periodontitis and T2D, single cell-type expression analysis using single cell RNA-seq (scRNA-seq) data from GEO database was conducted (Fig. 6A). Cells were clustered into 24 clusters and were further classified into 5 cell types (monocyte, NK cell, B cell, T cell, platelet) (Fig. 6B-C). The 6 candidate genes were enriched in different cell types (Fig. 6D; Supplementary Fig. 5). The 2 risk genes, *RAP2A* and *MCUR1* were enriched in NK cells and platelets in PBMCs from patients (Fig. 6E-F).

## Discussion

Periodontitis patients have a higher risk of T2D that places substantial socioeconomic burdens on individuals and global health economies [44]. Previous studies focus on the impact of T2D on periodontitis, and this study is the first to leverage a multi-omics integration method to detect the role of periodontitis in T2D pathogenesis and the possible molecular mechanisms. These findings have significant implications for public health and global economy, emphasizing that more attention should be dedicated to periodontal health, as it is a highly modifiable factor that aids in the prevention and management of T2D.

Two-sample MR analysis genetically predicted that periodontitis was causally associated with higher risk of T2D. 79 common DEGs associated with both periodontitis and T2D were then identified in transcriptome data. The integration of GWAS with the eQTLs of these genes from the peripheral blood prioritized 6 genes, including *MCUR1*, *RAP2A*, *FOS*, *PANX1*, *NFIX* and *WNK1*, in the pathogenesis of periodontitis-related T2D. So far, there have been two MR analyses on the relationship between periodontitis and T2D. However, these studies did not exclude all SNPs related to confounders when assessing causality and insufficiently encompassed SNPs to validate causality, reducing the reliability of their results. The potential mechanisms were not further analyzed as well [45, 46]. Our study incorporated the GWAS data from authoritative databases, and the Phenoscanner V2 was used to exclude SNPs associated with confounding factors and outcomes, ensuring highly reliable outcomes [22]. Furthermore, multi-omic approaches and datasets were originally integrated to elucidate the underlying mechanisms by which periodontitis promoted T2D. As verified by our study, colocalization analysis emphasized the importance of *RAP2A* in the pathogenesis of T2D, and the scRNA-seq datasets indicated that *RAP2A* was enriched in NK cells, suggesting the potential pathogenic mechanisms.

The peripheral blood tissue is essential for us to characterize genetic effects on gene expression and understand the complicated etiology of periodontitis-related T2D. In the analysis of MR and colocalization, *RAP2A* was discovered to result in T2D occurrence. *RAP2A* encodes RAP2A that is a small G protein with GTP-enzyme activity and an important intracellular signal transducer [47]. Only a few studies investigated RAP2A, which mainly focused on the molecular mechanisms of RAP2A in tumor migration and invasion [48, 49]. The current study is the first to investigate the molecular function of *RAP2A* as a risk factor of T2D, and detect that the rs11065987 was positively associated with T2D risk via increasing the expression of *RAP2A* according to colocalization analysis. Furthermore, *RAP2A* may participate in the pathogenesis of periodontitis-related T2D by regulating NK cells, as it was highly expressed in NK cells of patients with two diseases.

*MCUR1* is an integral membrane protein that binds to MCU and regulates mitochondrial Ca^2+^ uptake [50]. Up-regulation of *MCUR1* disrupts mitochondrial Ca^2+^ cycling and has been identified to be associated with several diseases [51–53]. Evidence have shown that mitochondrial dysfunction was associated with diabetic periodontitis [54]. Findings from the MR and scRNA-seq of the current study suggested that the evaluated expression of *MCUR1* in platelets from PBMCs contributed to the pathogenesis of periodontitis-related T2D, indicating the key roles of mitochondrial dysfunction in promoting T2D. These two risk genes are promising pharmacological targets of periodontitis patients to prevent T2D that merit further exploration.

Another 4 candidates, *FOS*, *PANX1*, *NFIX* and *WNK1*, were identified from blood tissue and might have protective influence on T2D. However, *PANX1*, *NFIX* and *WNK1* were up-regulated in T2D patients, which seems to be controversial. Presumably, the up-regulation of *PANX1* and *WNK1* might be protective response to T2D, as both *PANX1* and *WNK1* expression positively regulate glucose uptake [55, 56]. Further studies are required to better elucidate the role of *NFIX* in T2D.

The strength of this investigation is that it used MR and colocalization analyses to estimate the causal effects with the advantages of large sample sizes, minimizing the risk of reverse causation and confounding bias. Multi-omic evidence was also involved to further provide insights into the mechanisms of potential pathogenic genes. Some limitations of this study warrant recognition. Firstly, only 17 SNPs were selected as IVs for periodontitis. The limited number of IVs may introduce bias into the analysis due to collinearity, weak instruments and potential canceling effects among the IVs. Secondly, as the full biological function of the IVs have not been completely elucidated, conclusions cannot be draw that there is no pleiotropy. Despite the use of the MR-Egger method to limit pleiotropy, it has its limitations, such as dependence on the weak instrument assumption, and sensitivity to outliers [28]. As a result, the conclusion drawn from MR-Egger method was not completely reliable. Thirdly, the interpretation of PPH4 in colocalization should be careful, as a low PPH4 may not indicate lacking evidence for colocalization in situations where PPH3 is low as well, which may be the result of limited statistical power [37]. Fourthly, a sex-specific MR analysis on periodontitis and T2D had not been conducted in the present study. Previous studies have indicated a higher prevalence of periodontitis in males compared to females, with the condition often being more severe in males [57, 58], suggesting that males may represent a higher-risk group. Sex-stratified analysis is instrumental in pinpointing subgroups that exhibit varied responses to the exposure, thereby facilitating the implementation of personalized interventions. Ultimately, generalizing the findings of this study to other populations may be inappropriate given its exclusive focus on individuals of European descent.

## Conclusions

In conclusion, this study used a multi-omics integration method to explore causality between periodontitis and T2D, and underly molecular mechanisms using multiple bioinformatics tools. MR analyses detected that periodontitis was causally associated with higher risks of T2D and insulin resistance. Furthermore, *MCUR1*, *RAP2A*, *FOS*, *PANX1*, *NFIX* and *WNK1* were verified to play important roles in the pathogenesis of periodontitis-related T2D, shedding light on the discovery of potential drug targets for periodontitis patients to prevent T2D.

### Electronic supplementary material

Below is the link to the electronic supplementary material.

Supplementary Material 1

## Data Availability

The datasets supporting the conclusions of this article are available in the following repositories: DIAGRAM Consortium (http://www.diagram-consortium.org/), the IEU OpenGWAS project (ieu-a-1090) (https://gwas.mrcieu.ac.uk/), FinnGen Release 9 (https://www.finngen.fi/fi), MAGIC (http://magicinvestigators.org/), eQTLGen Consortium (https://eqtlgen.org/), GEO database (GSE189005; GSE10334; GSE244515) (https://www.ncbi.nlm.nih.gov/geo/).

## Abbreviations

T2D: Type 2 diabetes
MR: Mendelian randomization
eQTL: Expression quantitative trait loci
SNP: Single nucleotide polymorphism
DEG: Differentially expressed genes
GO: Gene ontology
KEGG: Kyoto Encyclopedia of Genes and Genomes
PBMC: Peripheral blood mononuclear cell
GWAS: Genome-wide association study
IVW: Inverse-variance-weight
MCUR1: Mitochondrial calcium uniporter regulator 1
RAP2A: RAS-related protein Rap-2a
FOS: FBJ murine osteosarcoma viral oncogene homolog
NFIX: Nuclear factor IX
PANX1: Pannexin 1
WNK1: With no lysine-1
