# Supplementary material for: Impact of periodontitis on type 2 diabetes: a bioinformatic analysis
Source: BMC Oral Health. 2024 May 29;24:635. doi: 10.1186/s12903-024-04408-1 (PMC11137885; doi:10.1186/s12903-024-04408-1)

**Impact of Periodontitis on Type 2 Diabetes: a Bioinformatic Analysis**

Xindi Wei^1#^, BDS; Xiaomeng Zhang^1#^, PhD; Ruiying Chen^1^, PhD; Yuan Li^1^, MDS; Yijie Yang^1^, MDS; Ke Deng^2^, PhD; Zhengzhen Cai^1^, MDS; Hongchang Lai^1*^, PhD; Junyu Shi^1*^, PhD

^#^Xindi Wei and Xiaomeng Zhang contributed equally as co-first authors.

^1^Department of Oral and Maxillofacial Implantology, Shanghai PerioImplant Innovation Center, Shanghai Ninth People's Hospital, Shanghai Jiao Tong University School of Medicine; College of Stomatology, Shanghai Jiao Tong University; National Center for Stomatology; National Clinical Research Center for Oral Diseases; Shanghai Key Laboratory of Stomatology; Shanghai Research Institute of Stomatology, Shanghai 200011, China

^2^Division of Periodontology and Implant Dentistry, The Faulty of Dentistry, The University of Hong Kong, Hong Kong 999077, China

**Supplementary material**

**Supplementary Table 1**. Details of the consortia from which IVs were selected.

**Supplementary Table 2**. List of IVs utilized in the MR analyses for the exposure of periodontitis.

**Supplementary Table 3**. Results of colocalization analyses for candidate genes.

**Supplementary Figure 1**. Sensitivity analyses for the exposure of periodontitis on T2D (discovery phase). The (A) IVW leave-one-out analysis, (B) scatter plot, (C) forest plot, and (D) funnel plot for the exposure of periodontitis and the outcome of T2D (DIAGRAM).

**Supplementary Figure 2**. Sensitivity analyses for the exposure of periodontitis on T2D (validation phase). The (A) IVW leave-one-out analysis, (B) scatter plot, (C) forest plot, and (D) funnel plot for the exposure of T2D and the outcome of periodontitis (UK Biobank).

**Supplementary Figure 3**. Sensitivity analyses for the exposure of periodontitis on FI. The (A) IVW leave-one-out analysis, (B) scatter plot, (C) forest plot, and (D) funnel plot for the exposure of periodontitis and the outcome of T2D.

**Supplementary Figure 4**. Colocalization analyses of *WNK1*, *NFIX*, *FOS* and *PANX1*. (A) The regional association plot for colocalization analysis of *WNK1*. (B) The regional association plot for colocalization analysis of *NFIX*. (C) The regional association plot for colocalization analysis of *FOS*. (D) The regional association plot for colocalization analysis of *PANX1.*

**Supplementary Figure 5**. Single cell-type expression of *WNK1, NFIX, FOS* and *PANX1* in PBMCs. (A) The expression of *WNK1* in each cell type. (B) The expression of *NFIX* in each cell type. (C) The expression of *FOS* in each cell type. (D) The expression of *PANX1* in each cell type.

**Supplementary Table 1. Details of the consortia from which IVs were selected.**

| **Phenotype** | **Consortium** | **Ancestry** | **Cases** | **Controls** | **Link** |
| --- | --- | --- | --- | --- | --- |
| Periodontitis | FinnGen R9 | European | 4,434 | 259,234 | https://www.finngen.fi/fi |
| T2D | DIAGRAM | European | 180,834 | 1,159,055 | http://www.diagram-consortium.org/ |
| T2D | IEU OpenGWAS | European | 4,040 | 116,246 | http://gwas.mrcieu.ac.uk/datasets/ieu-a-1090/ |
| FI | MAGIC | European | 281,416 |  | http://magicinvestigators.org/ |
| eQTL | eQTLGen | European | 14,263 |  | https://www.eqtlgen.org/ |

**Supplementary Table 2. List of IVs utilized in the MR analyses for the exposure of periodontitis.**

| **other_allele** | **effect_allele** | **SNP** | **pval** | **beta** | **se** | **eaf** | **F_statistics** |
| --- | --- | --- | --- | --- | --- | --- | --- |
| C | T | rs1148464 | 3.25E-06 | -0.143012 | 0.0307265 | 0.866449 | 21.66284436 |
| C | T | rs115120340 | 3.01E-07 | 0.184889 | 0.0360905 | 0.0917732 | 26.24418286 |
| A | G | rs139232605 | 1.13E-07 | 0.221301 | 0.041721 | 0.061807 | 28.13546128 |
| G | A | rs7629105 | 3.26E-06 | -0.189193 | 0.0406564 | 0.933376 | 21.65454148 |
| C | T | rs6845106 | 3.02E-06 | -0.101651 | 0.0217698 | 0.59251 | 21.80274313 |
| A | G | rs113059383 | 2.88E-06 | -0.839874 | 0.179505 | 0.00607947 | 21.89131648 |
| G | A | rs146734691 | 4.83E-06 | 0.733419 | 0.160408 | 0.00276423 | 20.9049422 |
| A | G | rs10268587 | 2.10E-06 | -0.294678 | 0.0621204 | 0.0368634 | 22.50213085 |
| G | A | rs141098993 | 2.25E-06 | 0.268631 | 0.0567977 | 0.0315562 | 22.36904609 |
| G | T | rs2847728 | 3.33E-06 | -0.125939 | 0.027086 | 0.210785 | 21.61859365 |
| T | C | rs11605185 | 1.12E-06 | -0.23119 | 0.0474857 | 0.0612053 | 23.70331603 |
| G | A | rs1241497 | 3.63E-06 | -0.127531 | 0.0275344 | 0.816954 | 21.45245509 |
| T | A | rs72682016 | 2.89E-06 | -0.21705 | 0.0463932 | 0.0663037 | 21.88807799 |
| A | T | rs56265851 | 1.63E-06 | 0.236029 | 0.0492277 | 0.0443149 | 22.98837897 |
| T | A | rs2220232 | 1.45E-06 | 0.103497 | 0.0214795 | 0.454101 | 23.21688195 |
| A | G | rs35813112 | 1.79E-06 | 0.235956 | 0.0494027 | 0.042846 | 22.81168607 |
| C | T | rs130985 | 4.77E-06 | -0.313082 | 0.0684394 | 0.0297213 | 20.92668888 |

**Supplementary Table 3. Results of colocalization analyses for candidate genes.**

|  | **PP. H0. abf** | **PP. H1. abf** | **PP. H2. abf** | **PP. H3. abf** | **PP. H4. abf** |
| --- | --- | --- | --- | --- | --- |
| ***RAP2A*** | 7.09e-03 | 1.38e-04 | 3.55e-01 | 6.26e-03 | 6.32e-01 |
| ***MCUR1*** | 2.265-05 | 7.32e-01 | 2.12e-08 | 4.17e-04 | 2.68e-01 |
| ***FOS*** | 3.59e-18 | 8.25e-01 | 6.84e-20 | 1.56e-02 | 1.59e-01 |
| ***WNK1*** | 2.31e-24 | 1.06e-24 | 4.79e-01 | 2.19e-01 | 3.02e-01 |
| ***NFIX*** | 3.13e-07 | 7.39e-01 | 9.58e-10 | 2.00e-03 | 2.59e-01 |
| ***PANX1*** | 4.89e-304 | 1.96e-304 | 4.49e-01 | 1.79e-01 | 3.72e-01 |

**Supplementary Figure 1. Sensitivity analyses for the exposure of periodontitis on T2D (discovery phase)** The (A) IVW leave-one-out analysis, (B) scatter plot, (C) forest plot, and (D) funnel plot for the exposure of periodontitis and the outcome of T2D.


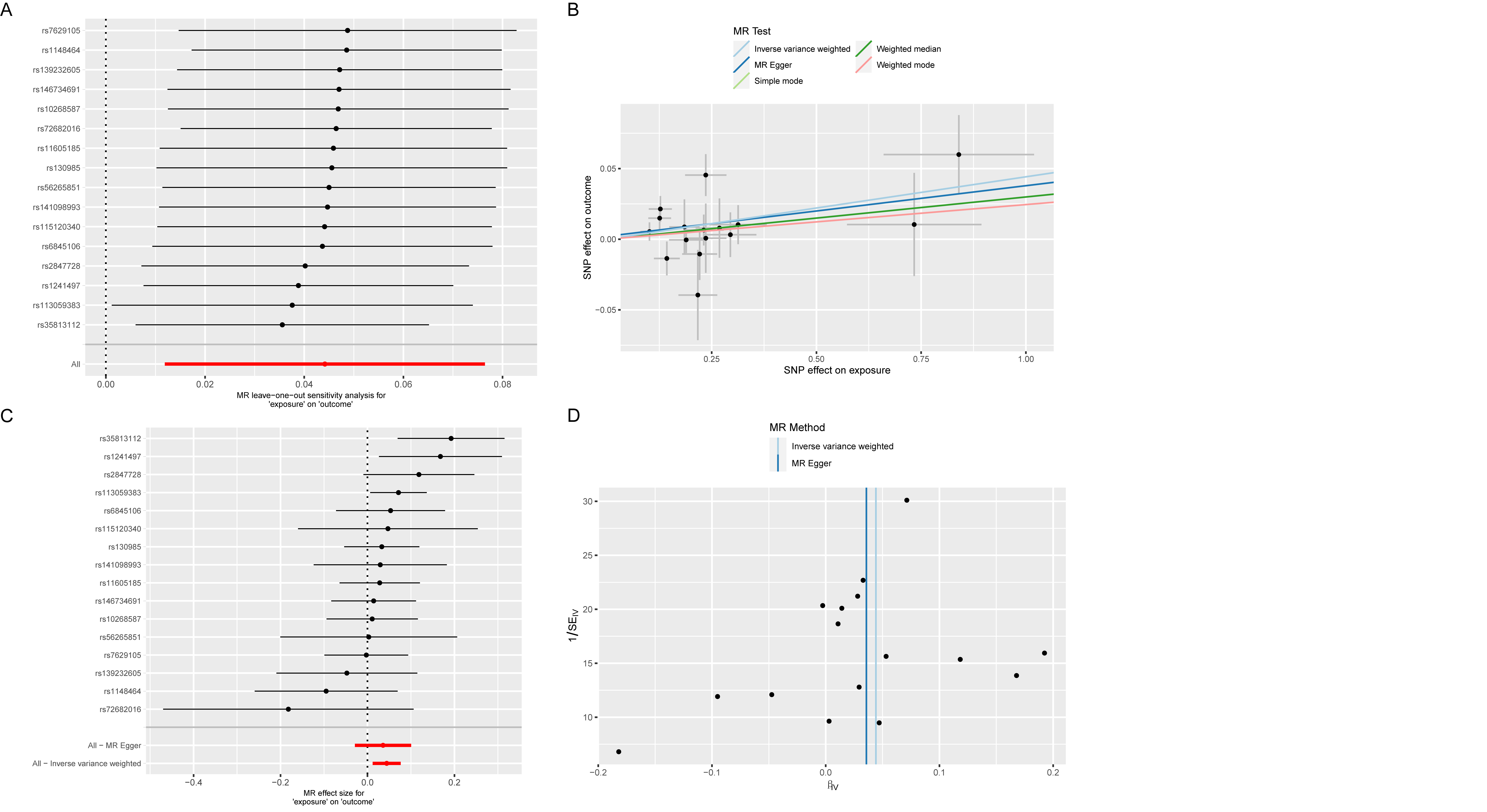


**Supplementary Figure 2. Sensitivity analyses for the exposure of periodontitis on T2D (validation phase).** The (A) IVW leave-one-out analysis, (B) scatter plot, (C) forest plot, and (D) funnel plot for the exposure of T2D and the outcome of periodontitis (UK Biobank).


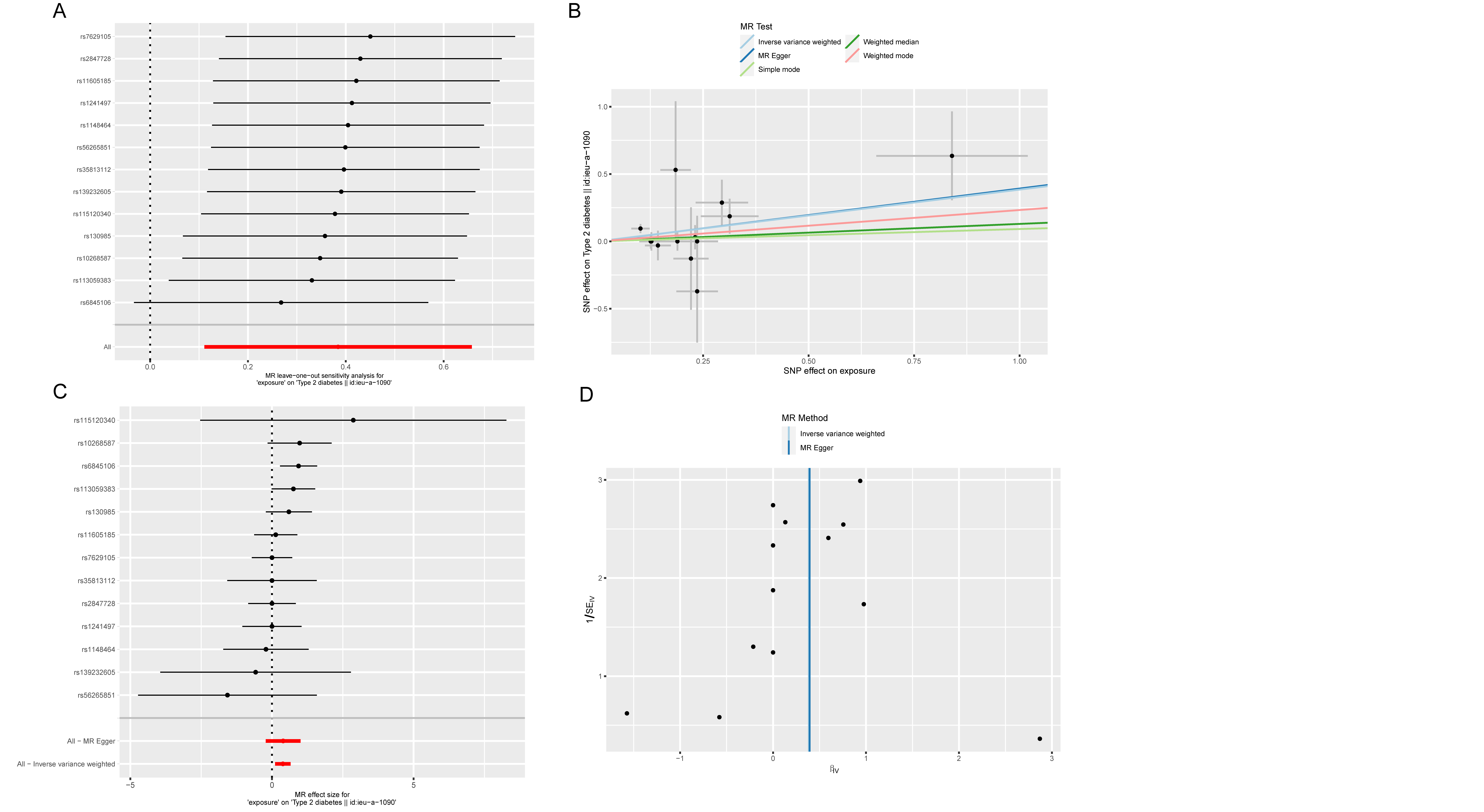


**Supplementary Figure 3. Sensitivity analyses for the exposure of periodontitis on FI.** The (A) IVW leave-one-out analysis, (B) scatter plot, (C) forest plot, and (D) funnel plot for the exposure of periodontitis and the outcome of T2D.


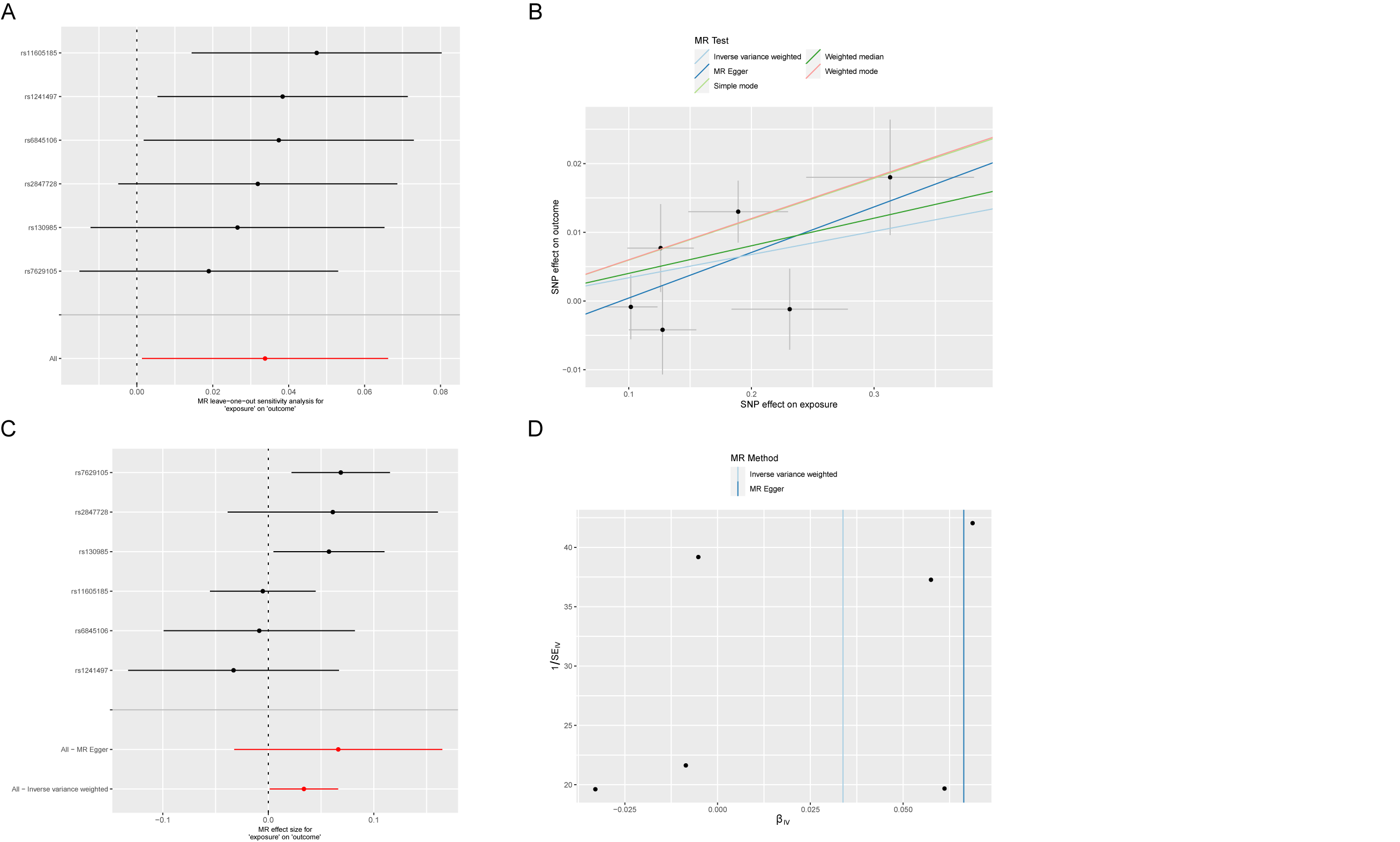


**Supplementary Figure 4. Colocalization analyses of *WNK1*, *NFIX*, *FOS* and *PANX1*.** (A) The regional association plot for colocalization analysis of *WNK1*. (B) The regional association plot for colocalization analysis of *NFIX*. (C) The regional association plot for colocalization analysis of *FOS*. (D) The regional association plot for colocalization analysis of *PANX1.*


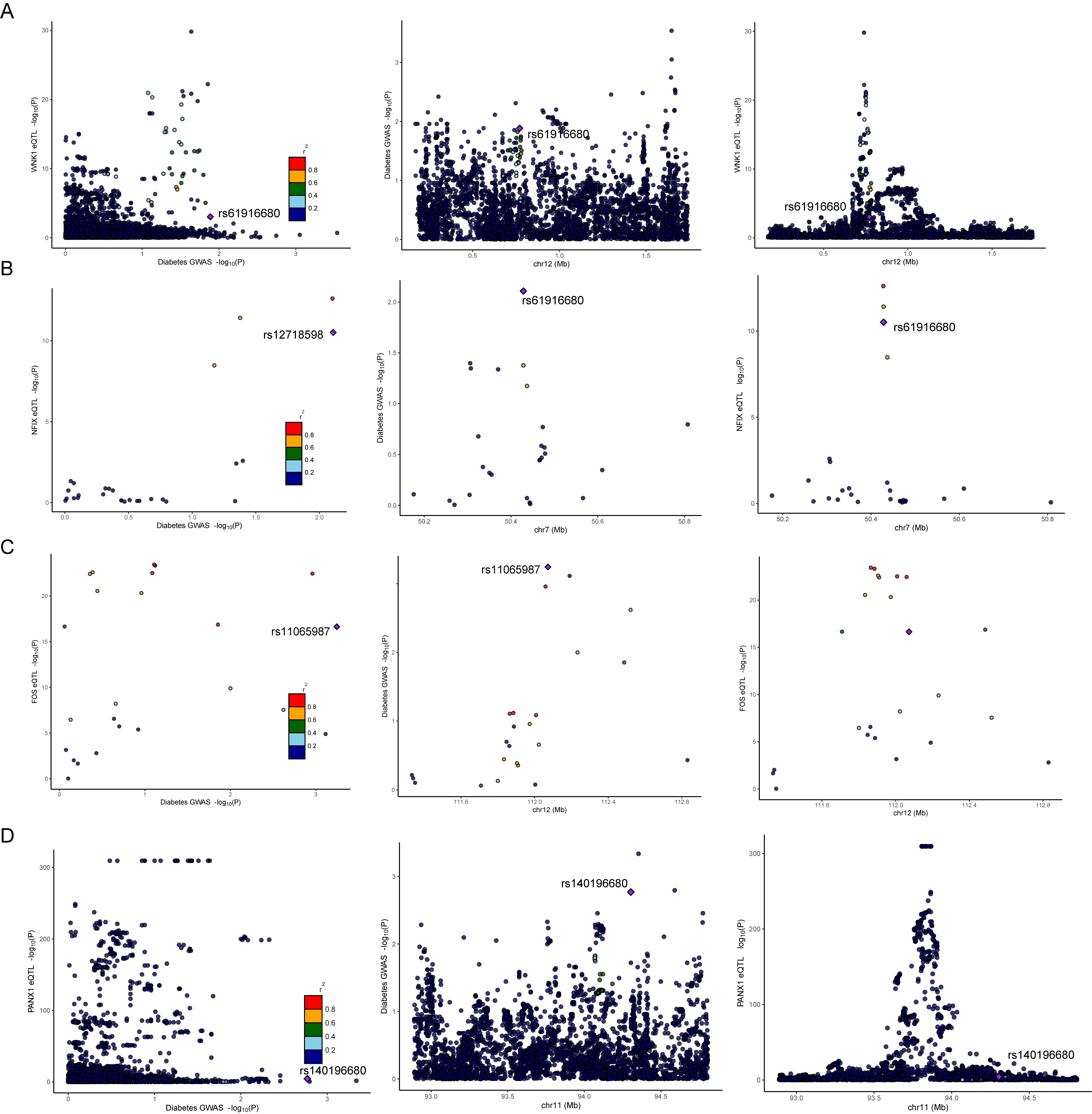


**Supplementary Figure 5. Single cell-type expression of *WNK1, NFIX, FOS* and *PANX1* in PBMCs.** (A) The expression of *WNK1* in each cell type. (B) The expression of *NFIX* in each cell type. (C) The expression of FOS in each cell type. (D) The expression of *PANX1* in each cell type.


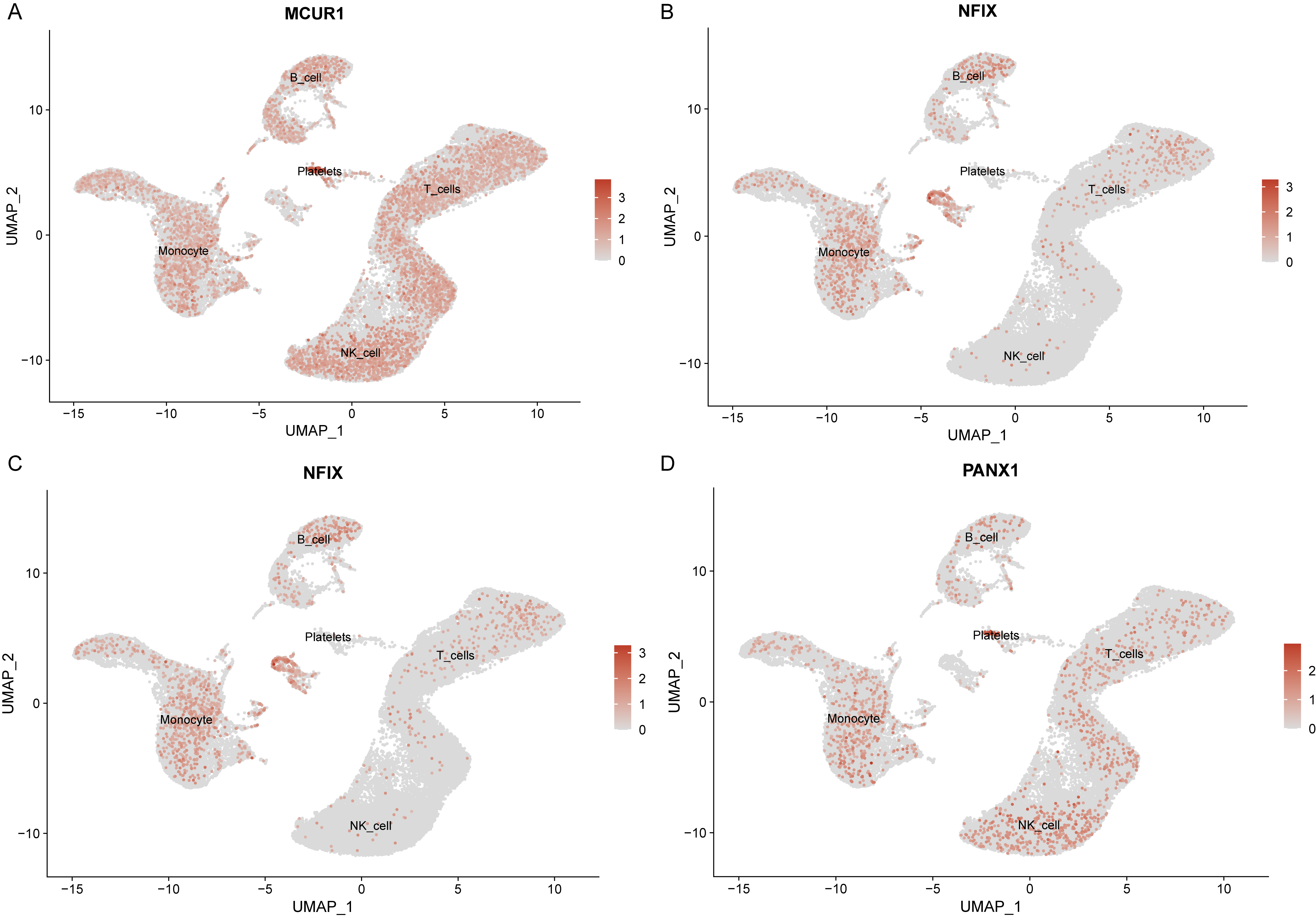

Supplement: Supplementary file 1 — Supplementary Material 1 [file 12903_2024_4408_MOESM1_ESM.docx]
